# Quality control of next-generation sequencing data without a reference

**DOI:** 10.3389/fgene.2014.00111

**Published:** 2014-05-06

**Authors:** Urmi H. Trivedi, Timothée Cézard, Stephen Bridgett, Anna Montazam, Jenna Nichols, Mark Blaxter, Karim Gharbi

**Affiliations:** ^1^Edinburgh Genomics, Ashworth Laboratories, University of EdinburghEdinburgh, UK; ^2^Institute of Evolutionary Biology, Ashworth Laboratories, University of EdinburghEdinburgh, UK

**Keywords:** Illumina sequencing, *de novo* assembly, quality control, insert size, PCR duplicates, mate pair

## Abstract

Next-generation sequencing (NGS) technologies have dramatically expanded the breadth of genomics. Genome-scale data, once restricted to a small number of biomedical model organisms, can now be generated for virtually any species at remarkable speed and low cost. Yet non-model organisms often lack a suitable reference to map sequence reads against, making alignment-based quality control (QC) of NGS data more challenging than cases where a well-assembled genome is already available. Here we show that by generating a rapid, non-optimized draft assembly of raw reads, it is possible to obtain reliable and informative QC metrics, thus removing the need for a high quality reference. We use benchmark datasets generated from control samples across a range of genome sizes to illustrate that QC inferences made using draft assemblies are broadly equivalent to those made using a well-established reference, and describe QC tools routinely used in our production facility to assess the quality of NGS data from non-model organisms.

## Introduction

Until 5 years ago, genomic research was largely confined to a relatively small number of taxonomic groups in which sequencing efforts were focused on a handful of model organisms. Next-generation sequencing (NGS) technologies have expanded the scope of genomics research by increasing throughput many fold compared to traditional Sanger sequencing, at a much lower cost per base (Pareek et al., 2011) With genome-scale studies now possible in virtually any species within the budget of a standard grant, NGS data are being generated in non-model organisms at an unprecedented pace. However, NGS can be affected by a range of artifacts that arise during the library preparation and sequencing processes, which can negatively impact the quality of the raw data for downstream analyses. These issues include platform specific error profiles, systematic variation in quality scores across the sequence read, biases in sequence generation driven by base composition, departure from optimal library fragment sizes, variation in the proportions of duplicate sequences introduced by PCR amplification bias, and contamination from known and unknown species other than the sequencing target (Schmieder and Edwards, 2011a; Zhou et al., 2013).

Several software tools have been published that can highlight quality issues in NGS data, including low base quality, contamination with adapter sequences and biases in base composition (e.g., Andrews, 2010; Lohse et al., 2012; Patel and Jain, 2012). Initial steps in the quality control (QC) process typically involve assessing the intrinsic quality of the raw reads using metrics generated by the sequencing platform (e.g., quality scores) or calculated directly from the raw reads (e.g., base composition). One of the most popular tools for the generation of these quality metrics is FastQC (http://www.bioinformatics.babraham.ac.uk/projects/fastqc/). FastQC and other similar tools are useful for assessing the overall quality of a sequencing run and are widely used in NGS data production environments as an initial QC checkpoint. Further QC steps commonly performed involve mapping the raw reads to a known reference to calculate a range of metrics from alignment profiles. These include the mapping rate to the expected target, levels of fragment or sequence duplication, and estimates of the library insert sizes. These metrics are routinely calculated for NGS data derived from model organisms where a well-established reference is available and generally included in QC reports. However this alignment-based approach is not directly possible when sequencing a novel genome. Tools exist that can calculate QC metrics such as sequencing errors and over-represented sequences in k-mer space without a reference genome (Schroder et al., 2010; Keegan et al., 2012; Wang et al., 2012). However, these do not generally predict library insert size and duplication rate. The preqc component of SGA (Simpson and Durbin, 2011; Simpson, 2014) can predict genome characteristics and QC metrics including fragment length and duplication levels but as these metrics are calculated only on a subset of the data in k-mer space, duplicate rate for a large dataset can be massively underestimated. Also, estimating insert size for mate pair libraries is not practical with this approach. Other tools including PRINSEQ (Schmieder and Edwards, 2011b), FASTX-Toolkit^1^ (http://hannonlab.cshl.edu/fastx_toolkit/), and CD-HIT (Fu et al., 2012) can predict the rate of fragment or read duplication without a reference, but have significant limitations. As these techniques are based on sequence-clustering algorithms, identical sequences, which might or might not be duplicates, can be erroneously removed. In addition, these approaches are both time consuming and computer memory intensive, and can create bottlenecks in a high throughput production environment where rapid and efficient QC of raw NGS data is necessary.

Detecting contaminants in the absence of a reference is equally challenging. Published methods exist for the detection of read contaminants, e.g., DeconSeq (Schmieder and Edwards, 2011a) and FastQ Screen (Andrews, 2011). These tools are based on identification of contamination from known sources by optimized alignment methods. However they fail when the sequence of the contaminant is not present in the screening database. Similarly, BLAST-based methods are computationally expensive when applied to large raw read datasets and cannot be implemented in a production environment.

In this study, we show how it is possible to generate a draft assembly from the raw data, rapidly and without optimization, and then use this for the generation of reliable QC metrics. To illustrate the utility of this approach, we generated benchmark sequence datasets from control samples of three model species (*Escherichia coli*, *Arabidopsis thaliana* and *Homo sapiens*), for which a high quality reference sequence is available, and applied our QC tools to the raw reads. By employing both standard mapping-based tools to estimate PCR duplicate rates and library insert sizes, and new approaches such as the taxon-annotated GC-coverage (TAGC) plot pipeline (Kumar et al., 2013) to identify contaminants, we show broad equivalence of the *de novo* and reference-based QC approaches.

## Materials and methods

### Library preparation and sequencing of control samples

DNA and RNA samples used to generate control libraries were obtained from commercial sources (*E. coli* K12 DNA: Invivogen, catalog no. tlrl-ednaef; *H. sapiens* DNA: Coriell Institute for Medical Research, catalog no. NA10857; *A. thaliana* DNA: AMS Biotechnology, catalog no. D1634310; *H. sapiens* RNA: Ambion, catalog no. AM7962). All samples were quantified by fluorescence-based measurements (Invitrogen Qubit) and assessed for quality using Life Technologies E-gels (DNA) or Agilent Technologies Bioanalyzer (RNA) before library preparation.

Genomic libraries with insert sizes of 180, 300, and 600 bp were prepared for all three species using Illumina TruSeq DNA Sample Prep Kit following the manufacturer's instructions with some modifications. Briefly, 3 μg of genomic DNA was sheared using a Covaris S2 instrument (180 bp: duty cycle 10%, intensity 5, cycles/burst 200, time 420 s; 300 bp: duty cycle 10%, intensity 4, cycles/burst 200, time 110 s; 600 bp: duty cycle 5%, intensity 3, cycles/burst 200, time 80 s) in 120 μl reactions with 1X TE buffer, cleaned up with 1:1 ratio Ampure XP beads (Beckman Coulter Inc.), and ligated to unbarcoded Illumina paired-end adapters. Post-ligation, each library was individually size selected to the target size with a Sage Science BluePippin DNA size selection system using the 1.5% agarose gel cassette protocol and tight cuts at 320 bp (180 bp insert), 440 bp (300 bp insert) and 740 bp (600 bp insert). Size selected libraries were eluted in 40 μl volumes and enriched by PCR using library-specific indexed primers complementary to the Illumina paired-end adapters.

The *E. coli* mate-pair library was constructed using a combination of Life Technologies SOLiD Long Mate-Paired Library Construction Kit and Illumina Mate Pair Library Prep Kit v2 following the manufacturers' recommendations.

*H. sapiens* transcriptome (RNAseq) libraries were prepared using Illumina TruSeq RNA Sample Prep Kit v2 following the manufacturer's instructions, using 1 μg total RNA input and 12 PCR cycles in the enrichment step.

The mock-contaminated library was created by spiking the 300 bp insert *E. coli* library (Eco300) into the 300 bp insert *H. sapiens* library (Hsa300) in proportions 1:20.

All libraries were checked on a Bioanalyzer High Sensitivity DNA Chip (Agilent Technologies) and quantified by qPCR (Kapa Library Quantification Kit) before Illumina sequencing on GAIIx, HiSeq 2500 or MiSeq platforms as per the manufacturer's instructions. Summary of all libraries is given in Table 1. Raw sequence data were submitted to the Short Read Archive with accession number ERP004578 (http://www.ebi.ac.uk/ena/data/view/ERP004578).

**Table 1:** **Summary of benchmark datasets**.

**Library**	**Species**	**Source**	**#Reads**	**#Bases (*Gb*)**	**#Coverage (*X*)**	**Insert size (*bp*)**	**Platform**	**Reads**
**GENOMIC LIBRARIES**								
Eco180	*Escherichia coli*	Invivogen	2,823,400	0.28	70.59	180	MiSeq	100PE (v2)
Eco300	*Escherichia coli*	Invivogen	3,325,790	0.33	83.14	300	MiSeq	100PE (v2)
Eco600	*Escherichia coli*	Invivogen	3,369,406	0.34	84.24	600	MiSeq	100PE (v2)
Eco3K	*Escherichia coli*	Invivogen	17,479,364	1.75	436.98	2500	GAIIx	100PE (v5)
Ath180	*Arabidopsis thaliana*	Amsbio	59,777,644	5.98	46.7	180	HiSeq 2500	100PE (rapid)
Ath300	*Arabidopsis thaliana*	Amsbio	72,813,974	7.28	56.89	300	HiSeq 2500	100PE (rapid)
Ath600	*Arabidopsis thaliana*	Amsbio	58,823,560	5.88	45.96	600	HiSeq 2500	100PE (rapid)
Hsa180	*Homo sapiens*	Coriell	318,394,090	31.84	10.61	180	HiSeq 2500	100PE (rapid)
Hsa300	*Homo sapiens*	Coriell	319,230,116	31.92	10.64	300	HiSeq 2500	100PE (rapid)
Hsa600	*Homo sapiens*	Coriell	255,145,408	25.51	8.5	600	HiSeq 2500	100PE (rapid)
EH-mock	*Homo sapiens and Escherichia coli*	Hsa300 Eco300	283,522,600	28.35	8.96	300	HiSeq 2500	100PE (rapid)
**TRANSCRIPTOME LIBRARIES**								
HsaRNA1	*Homo sapiens*	Ambion	111,953,730	11.2	44.50	200	HiSeq 2500	100PE (rapid)
HsaRNA2	*Homo sapiens*	Ambion	99,968,630	10	39.74	201	HiSeq 2500	100PE (rapid)
HsaRNA3	*Homo sapiens*	Ambion	79,656,332	7.97	31.66	202	HiSeq 2500	100PE (rapid)

### Bioinformatics analyses

#### Pre-processing of reads

All reads were trimmed for adapter sequences and poor quality bases (<Q30) using fastq-mcf (http://code.google.com/p/ea-utils) with the following parameters *q* = 30, *l* = 35 and qual-mean = 30.

#### Mapping to reference

Reads were aligned to draft assemblies and reference genomes/transcriptomes using BWA 0.6.1 (Li and Durbin, 2009a,b) with default parameters. Insert size and PCR duplication rate metrics were obtained using PICARD^2^ (v.1.99) and alignment rate was calculated using SAMtools (v.0.1.18). The genomes of *E. coli* K12 MG1655 (http://www.ncbi.nlm.nih.gov/nuccore/NC_000913.3), *A. thaliana* TAIR10 (ftp://ftp.arabidopsis.org/home/tair/Sequences/whole_chromosomes/), and *H. sapiens* hg19 (http://hgdownload.soe.ucsc.edu/goldenPath/hg19/bigZips/) were used as references for mapping of reads derived from the relevant genomic libraries. For transcriptome data, mRNA sequences from UCSC were used as a second reference (along with the genome) to compare QC results using genomic *versus* transcriptomic references.

#### Contig assembly

We generated genome assemblies from genomic data using CLC Assembly Cell^3^ (v.4.2.0, thereafter referred to as CLC) and SOAPdenovo2 (Luo et al., 2012), and transcriptome assemblies from mRNA reads using CLC and SOAPdenovo-Trans (Xie et al., 2013). Paired-end and mate-pair data were treated as single-end data by combining both reads in a single file.

Two parameters were defined for SOAPdenovo2 and SOAPdenovo-Trans: k-mer size (K) was set to 31 and the minimum contig length cutoff was set to 100. The choice of k-mer was not optimized, as our aim was to assemble reads into longer contigs and not to generate the best assembly. By default SOAPdenovo2 reports contigs with minimum length cutoff of K^*^2, but we observed that very small contigs (62 bases, if *K* = 31) were too short for the QC analyses we wanted to perform. No parameter optimization was used for CLC because the program estimates optimal parameters based on the data.

Two quality metrics were calculated to describe draft assemblies: % assembly size (the proportion of the reference covered by the draft assembly) and % chaff contig size (the proportion of the assembly made up of contigs less than or equal to 300 bases) (Salzberg et al., 2012).

#### Contamination check

The proportion of G and C bases (GC content) and the read coverage for each contig in the draft assembly of this mixed dataset were calculated using the TAGC plot pipeline (available at https://github.com/sujaikumar/assemblage; Kumar and Blaxter, 2011; Kumar et al., 2013). To identify potential contaminants *de novo*, contigs or a subset of contigs from the assemblies of the genomic data were compared to the National Center for Biotechnology Information (NCBI) non-redundant nucleotide database (nt) using megablast program in BLAST (ncbi-blast-2.2.28+) (Altschul et al., 1990). The hits obtained were then used to generate TAGC plots (Kumar et al., 2013), which were reviewed manually.

## Results

### Overview of QC assemblies

We generated draft QC assemblies for each library using CLC, which we used in-house in our QC pipeline, and another, open-source assembler, SOAPdenovo2, for comparison. Detailed metrics of all the assemblies are given in Table 2.

**Table 2:** **Assembly metrics**.

**Library**	**Assembler**	**Max contig length (*bp*)**	**#Contigs**	**Total bases (*Mb*)**	**N50 (bp)**	**GC contigs (%)**	**#Contigs >1 kb**	**Total bases in contigs >1 kb (Mb)**	**GC contigs >1 kb (%)**	**Chaff size (%)**	**Assembly size (%)**
**GENOMIC LIBRARIES**											
Eco180	CLC	326,302	151	4.47	82,998	50.7	116	4.45	50.7	0.07	96.51
Eco180	SOAPdenovo2	25,722	3569	4.67	4555	50.5	1082	4.23	50.8	5.08	100.29
Eco300	CLC	286,650	183	4.47	76,328	50.7	122	4.45	50.7	0.11	96.67
Eco300	SOAPdenovo2	21,043	5411	4.84	3646	50.2	1236	4.12	50.7	8.91	103.84
Eco600	CLC	326,302	168	4.47	78,532	50.7	116	4.45	50.7	0.1	96.6
Eco600	SOAPdenovo2	9871	12,296	5.34	1406	50	1632	3.31	50.7	21.35	114.64
Eco3K	CLC	171,461	246	4.45	43,133	50.7	202	4.43	50.7	0.09	96.23
Eco3K	SOAPdenovo2	127,995	589	4.46	18,364	50.7	378	4.4	50.7	0.53	95.78
Ath180	CLC	132,653	40,865	116.41	14,227	37.5	13,208	104.71	36.5	1.8	97.68
Ath180	SOAPdenovo2	45,556	643,869	170.25	910	43.8	24,315	83.44	36.4	57.62	141.99
Ath300	CLC	184,969	37,278	120.34	16,119	37.6	13,493	110	36.9	1.55	100.97
Ath300	SOAPdenovo2	51,622	976,946	202.46	392	44.7	26,270	85.71	37	81.6	168.82
Ath600	CLC	152,702	35,436	112.8	15,306	36.8	11,836	103.19	36.1	1.71	94.65
Ath600	SOAPdenovo2	41,984	714,501	170.19	826	42.5	24,038	82.01	36.2	59.85	141.92
Hsa180	CLC	42,593	2,212,575	2237.36	1724	40.4	665,315	1524.53	41	3.22	72.07
Hsa180	SOAPdenovo2	23,451	3,966,705	2297.79	1185	39.9	664,849	1291.64	40.7	11.02	73.57
Hsa300	CLC	42,643	2,135,062	2173.2	1720	40.6	648,840	1484.65	41.2	3.07	70
Hsa300	SOAPdenovo2	33,412	4,179,352	2291	1141	39.9	649,236	1255.44	41	11.95	73.35
Hsa600	CLC	42,628	2,465,124	1639.48	926	42.1	424,045	781.62	44.3	5.14	52.83
Hsa600	SOAPdenovo2	13,924	6,048,329	2029.43	570	40.8	402,649	673.79	44.3	20.62	65
**TRANSCRIPTOME LIBRARIES**											
HsaRNA1	CLC	14,039	114,337	79.71	1069	46.8	18,807	41.13	47	1.69	16.12
HsaRNA1	SOAPdenovo-TRANS	14,350	346,228	106.18	587	46.3	19,740	41.68	47.4	7.63	21.36
HsaRNA2	CLC	16,645	98,113	69.55	1077	47.4	16,762	36.04	47.7	1.34	14.06
HsaRNA2	SOAPdenovo-TRANS	12,835	228,311	84.44	747	47.2	17,674	36.78	48.1	5.04	16.98
HsaRNA3	CLC	16,591	106,392	73.95	1069	46.9	17,574	38.17	47	1.6	14.95
HsaRNA3	SOAPdenovo-TRANS	12,198	339,888	100.75	559	46.3	18,579	38.69	47.5	7.5	20.27

*Values for Assembly size and Chaff size are expressed as a percentage of the true genome size*.

### *Escherichia coli* genomic data

CLC assembled the *E. coli* 180 bp insert (Eco180), 300 base pair insert (Eco300), 600 bp insert (Eco600) and 3 kb mate-pair (Eco3K) libraries into 151, 183, 168, and 246 contigs, respectively. Most contigs in each assembly were over 1 kb. SOAPdenovo2 consistently produced larger numbers of contigs: 3569 contigs for Eco180, 5411 contigs for Eco300, 12,296 contigs for Eco600, and 589 contigs for Eco3K.

### *Arabidopsis thaliana* genomic data

CLC assembled the *A. thaliana* reads into 40,865, 37,278, and 25,436 contigs from the 180, 300, and 600 bp insert libraries respectively, with fewer than 2% of bases in chaff contigs. SOAPdenovo2 produced 643,869, 976,946, and 714,501 contigs with 57.62, 81.60, and 59.85% of bases in chaff contigs (contigs <300 bp) for the 180 bp (Ath180), 300 bp (Ath300) and 600 bp (Ath600) libraries respectively.

### *Homo sapiens* genomic data

Both CLC and SOAPdenovo2 produced highly fragmented assemblies from the *H. sapiens* reads containing millions of contigs for each library. The chaff contig size proportion was higher for the SOAPdenvo2 assemblies (11–20%) than for the CLC assemblies (3–5%). The proportion of the genome assembled for all libraries was ~75%. Obviously, much greater coverage is required to generate full assembly representation of the 3 Gb human genome.

### *Homo sapiens* transcriptomic data

The *H. sapiens* RNAseq libraries were assembled using CLC and SOAPdenovo-TRANS. CLC generated fewer contigs (or transcript fragments; 114,337, 98,113, and 106,392 contigs for HsaRNA1, HsaRNA2, and HsaRNA3 respectively) than did SOAPdenovo-TRANS (346,228, 228,311, and 339,888 contigs for HsaRNA1, HsaRNA2, and HsaRNA3 respectively). The proportion of chaff contigs was relatively low for both assemblers: <2% for CLC assemblies and <8% for SOAPdenovo2 assemblies. The assembly size for both tools was ~20% of the UCSC mRNA reference, indicating significant incompleteness relative to the whole human transcriptome, but likely reflecting restricted gene expression in the tissue surveyed.

### Duplicate rate

#### Genomic libraries

**Figure 1:**
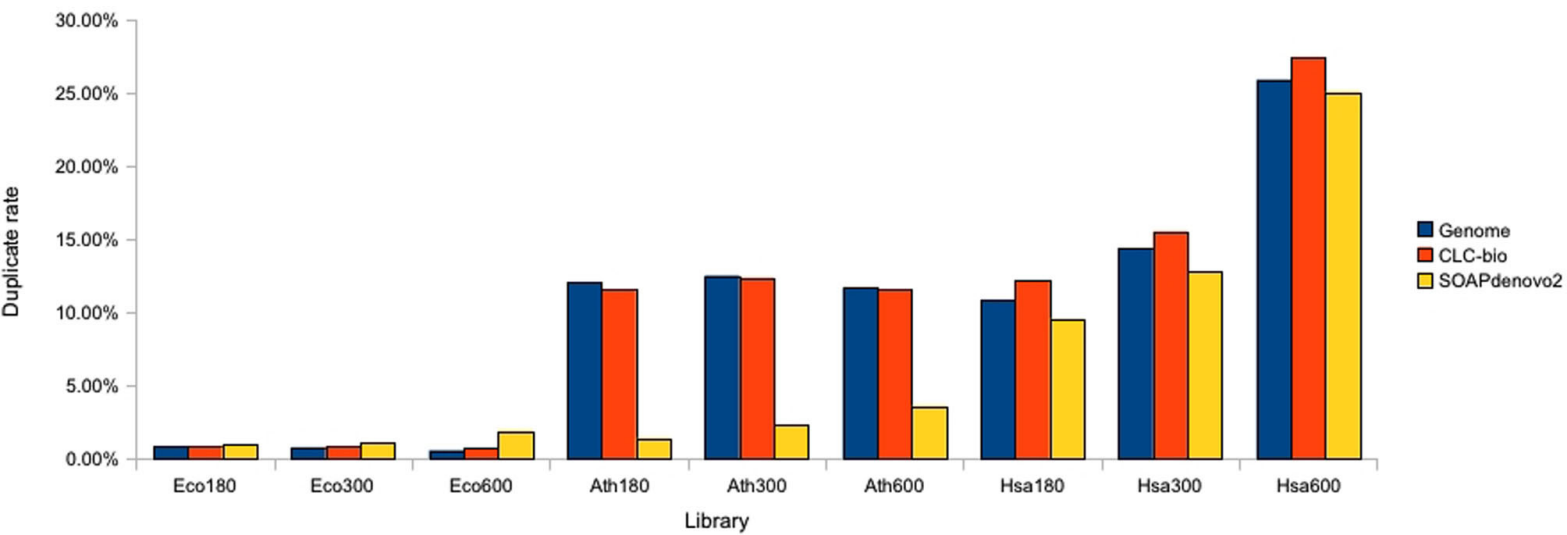
**Estimation of duplicate rate for paired-end genomic libraries**. Duplicate rates are plotted for each species and each target size using reads mapped against the species reference, CLC, and SOAPdenovo2 assemblies.

The mapping rate for the three *E. coli* libraries was 98% when mapped to the standard reference. Mapping to the draft CLC assembly produced a similar mapping rate. When the SOAPdenovo2 assembly was used as a reference the mapping rate was slightly reduced to 95% for the Eco180 and Eco300 libraries, and to 90% for the Eco600 library (Table 3, Figure 1). Fewer PCR duplicates were identified against the reference and the CLC assemblies than against the SOAPdenovo2 assemblies. The mapping rate for the *E.coli* 3 kb mate pair library was ~98% to the standard reference genome, the CLC assemblies and the SOAPdenovo2 assemblies, with consistent duplicate rates across methods (Table 3, Figure 1).

**Table 3:** **Mapping statistics for genomic libraries**.

**Sample ID**	**#Reads mapped**	**%Reads mapped**	**#Duplicate reads**	**%Duplicate reads**	**Mean insert length**	**SD insert length**
**ALIGNMENT TO REFERENCE GENOME**						
Eco180	2,792,154	98.89	22,999	0.82	176.59	21.44
Eco300	3,289,021	98.89	25,202	0.77	291.71	20.37
Eco600	3,321,671	98.58	18,691	0.56	573.11	70.04
Ecoli-3K	26,814,289	98.48	9,953,903	36.55	2720.28	315.33
Ath180	57,514,318	96.21	6,947,328	12.08	165.02	18.67
Ath300	69,958,509	96.08	8,749,750	12.51	293.22	27.48
Ath600	56,213,701	95.56	6,567,271	11.68	598.77	56.91
Hsa180	303,848,802	95.43	33,019,701	10.87	160.86	19.73
Hsa300	303,592,964	95.10	43,557,165	14.35	274.15	24.3
Hsa600	237,453,840	93.07	61,514,814	25.91	489.52	112.38
**ALIGNMENT TO CLC ASSEMBLY**						
Eco180	2,801,935	99.24	24,069	0.86	176.53	21.73
Eco300	3,301,631	99.27	27,735	0.84	291.42	20.8
Eco600	3,328,844	98.80	23,556	0.71	572.84	70.29
Ecoli-3K	26,843,812	98.59	10,347,907	38.00	2719.67	305.54
Ath180	54,747,819	91.59	6,319,279	11.54	164.56	19.01
Ath300	66,917,669	91.90	8,269,427	12.36	289.86	33.49
Ath600	52,667,137	89.53	6,087,843	11.56	594.36	71.53
Hsa180	254,730,241	80.00	30,966,836	12.16	159.64	20.13
Hsa300	257,834,392	80.77	40,049,368	15.53	268.47	30.5
Hsa600	184,136,326	72.17	50,589,333	27.47	453.27	135.32
**ALIGNMENT TO SOAPdenovo2 ASSEMBLY**						
Eco180	2,719,197	96.31	26,608	0.98	175.69	22.02
Eco300	3,185,522	95.78	35,419	1.11	289.55	22.76
Eco600	3,058,613	90.78	58,099	1.90	565.64	76.81
Ecoli-3K	26,696,300	98.05	10,338,237	37.96	2715.88	305.67
Ath180	35,381,329	59.19	492,038	1.39	163.44	19.01
Ath300	43,453,271	59.68	987,491	2.27	291.18	25.74
Ath600	34,367,686	58.43	1,233,130	3.59	599.55	47.13
Hsa180	212,773,751	66.83	20,335,478	9.56	158.82	19.74
Hsa300	214,568,006	67.21	27,419,943	12.78	268.52	29.82
Hsa600	158,562,678	62.15	39,751,153	25.07	466.71	124.63

The mapping rate for the *A. thaliana* libraries was ~96% when mapped to the standard reference genome. This was ~90% when mapped to the CLC assemblies but dramatically lower (~59%) when mapped to the SOAPdenovo2 assemblies. In addition, the duplicate rate was predicted to be ~12% for all three libraries using the standard reference genome and CLC assemblies, but only 2% when using the SOAPdenovo2 assemblies (Table 3, Figure 1). To investigate this discrepancy, we examined the Ath180 library data further. All reads which were marked as duplicates after mapping to the standard reference genome were extracted and mapped to the SOAPdenovo2 assembly: 95% of these reads remained unmapped against the SOAPdenovo2 assembly. We observed that the SOAPdenovo2 assemblies contains a large proportion of bases in chaff contigs indicating that there are many regions of the genome failing to assemble, thus fragmenting the assembly. This fragmentation is likely to cause an “edge-effect” when reads are aligned with BWA. Internally, BWA concatenates all reference sequences (contigs in our case) into one long, contiguous sequence and a read can be mapped to the junction of two adjacent reference sequences. In this case BWA will flag the read as unmapped (http://bio-bwa.sourceforge.net/). This leads to an apparent reduction in both the mapping rate and the duplicate rate through the exclusion of reads aligned to the edge of contigs in the calculations of the PCR duplicate rate. To test this, we altered the k-mer used by SOAPdenovo2 in order to assemble the reads into longer contigs. We used KmerGenie (Chikhi and Medvedev, 2013) to select optimized parameters for the assembly. This suggested using a k-mer size of 45 and coverage cutoff of 2. We ran SOAPdenovo2 again using these parameters, which produced an improved assembly with 146,503 contigs and an N50 of 5748 bases. Reads for Ath180 were mapped to this assembly, which yielded a 25% increase in the mapping rate and a 3 % increase in the duplicate rate. When we mapped reads which were flagged as duplicates, against the standard reference genome to the improved assembly, we also observed an increase in the mapping from 5% to 20% (i.e., 80% of these remained unmapped).

For the *H. sapiens* data, the mapping rate was 95% against the standard reference genome. This reduced to 80% against the CLC assemblies for Hsa180 and Hsa300. The mapping rate was 70% for Hsa600 against the CLC assembly. The duplicate rate for these data was ~25% when reads were aligned against the standard reference and the SOAPdenovo2 assemblies, and slightly higher (27%) against the CLC assembly (Table 3).

#### Transcriptome libraries

**Figure 2:**
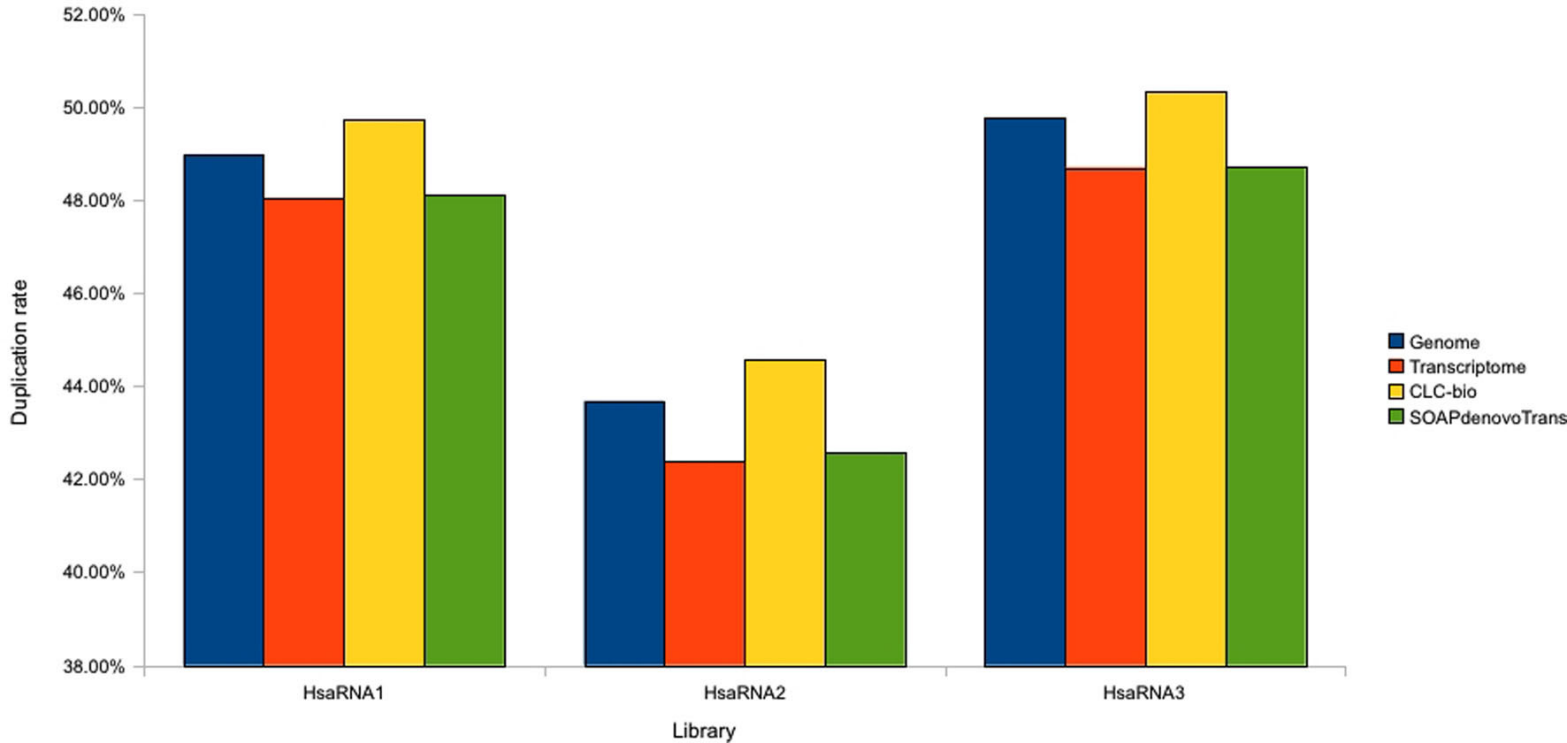
**Estimation of duplicate rate for transcriptomic libraries**. Duplicate rates are plotted for each replicate RNAseq library using reads mapped against the human genome and transcriptome references, CLC, and SOAPdenovo2 assemblies.

For the transcriptome (RNAseq) libraries, mapping results against the reference genome, reference transcriptome and the two assemblies were very similar (Table 4). The mapping rate was ~90% for alignment to the CLC assemblies and reference transcriptomes, and 94% for alignment to the SOAPdenovo-TRANS assemblies. The duplicate rate was consistent across all three mapping approaches for each replicate library (Figure 2). Some differences were observed between replicates, which can be attributed to differences in coverage (Table 1).

**Table 4:** **Mapping statistics for RNAseq libraries**.

**Sample ID**	**#Reads mapped**	**%Reads mapped**	**#Duplicate reads**	**%Duplicate reads**	**Mean insert length**	**SD insert length**
**ALIGNMENT TO GENOME hg19**						
HsaRNA1	102,189,446	91.28	50,803,861	49.72	215.99	72.03
HsaRNA2	72,036,569	90.43	32,114,542	44.58	184.88	64.15
HsaRNA3	91,195,551	91.22	45,907,384	50.34	211.94	67.64
**ALIGNMENT TO TRANSCRIPTOME**						
HsaRNA1	101,085,843	90.29	48,569,451	48.05	221.99	73.19
HsaRNA2	72,043,659	90.44	30,536,011	42.39	191.66	67.62
HsaRNA3	90,151,071	90.18	43,899,305	48.70	217.69	68.43
**ALIGNMENT TO CLC ASSEMBLY**						
HsaRNA1	102,553,145	91.60	50,224,113	48.97	214.16	67.59
HsaRNA2	72,815,405	91.41	31,802,909	43.68	184.07	62
HsaRNA3	91,194,129	91.22	45,376,410	49.76	210.12	63.27
**ALIGNMENT TO SOAPdenovo-TRANS ASSEMBLY**						
HsaRNA1	105,695,602	94.41	50,837,146	48.10	213.49	67.4
HsaRNA2	74,505,094	93.53	31,718,199	42.57	183.55	61.72
HsaRNA3	94,064,116	94.09	45,826,021	48.72	209.46	63.14

### Insert size distribution

**Figure 3:**
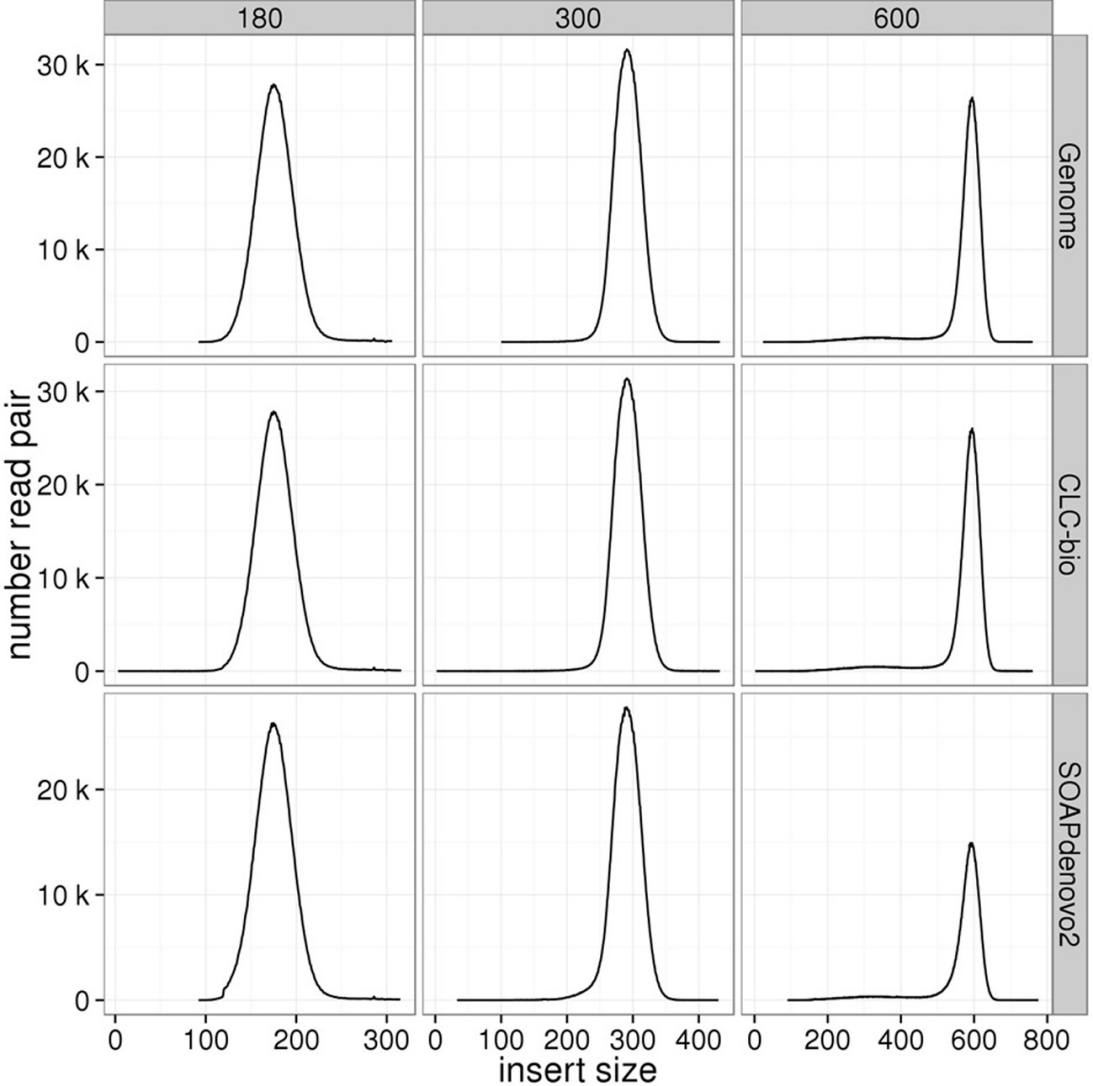
**Estimation of insert sizes for *E. coli* paired-end genomic libraries**. Insert sizes are plotted for each target size using reads mapped against the *E. coli* reference genome, CLC, and SOAPdenovo2 assemblies.

**Figure 4:**
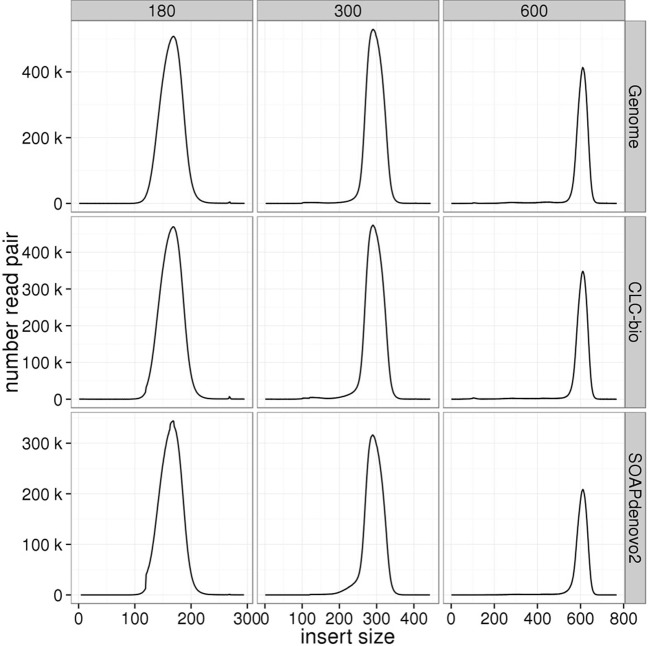
**Estimation of insert sizes for *A. thaliana* paired-end genomic libraries**. Insert sizes are plotted for each target size using reads mapped against the *A. thaliana* reference genome, CLC, and SOAPdenovo2 assemblies.

**Figure 5:**
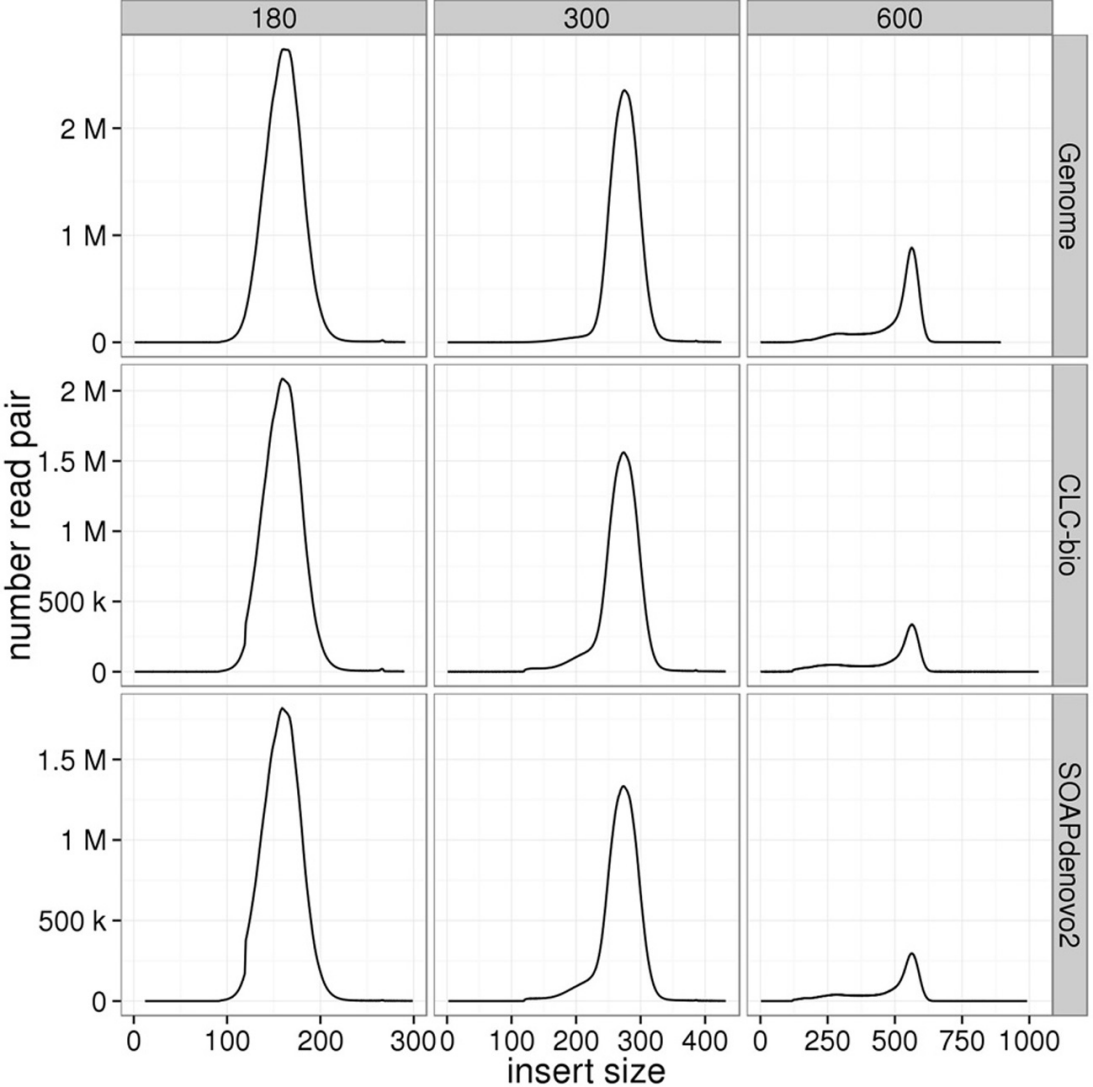
**Estimation of insert sizes for *H. sapiens* paired-end genomic libraries**. Insert sizes are plotted for each target size using reads mapped against the *H. sapiens* reference genome, CLC, and SOAPdenovo2 assemblies.

**Figure 6:**
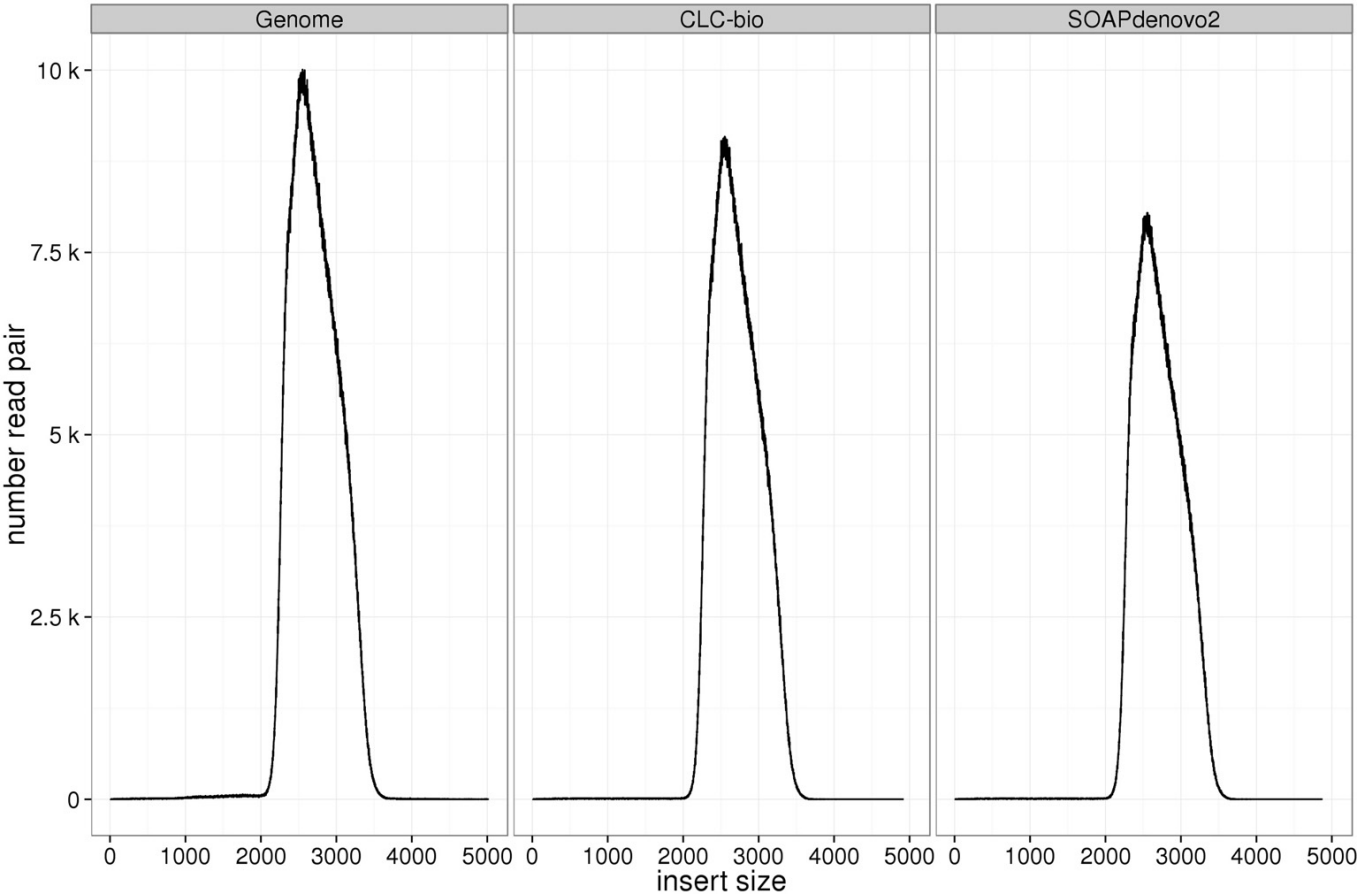
**Estimation of insert sizes for *E. coli* mate pair library**. Insert sizes are plotted using reads mapped against *E. coli* reference genome, CLC, and SOAPdenovo2 assemblies.

**Figure 7:**
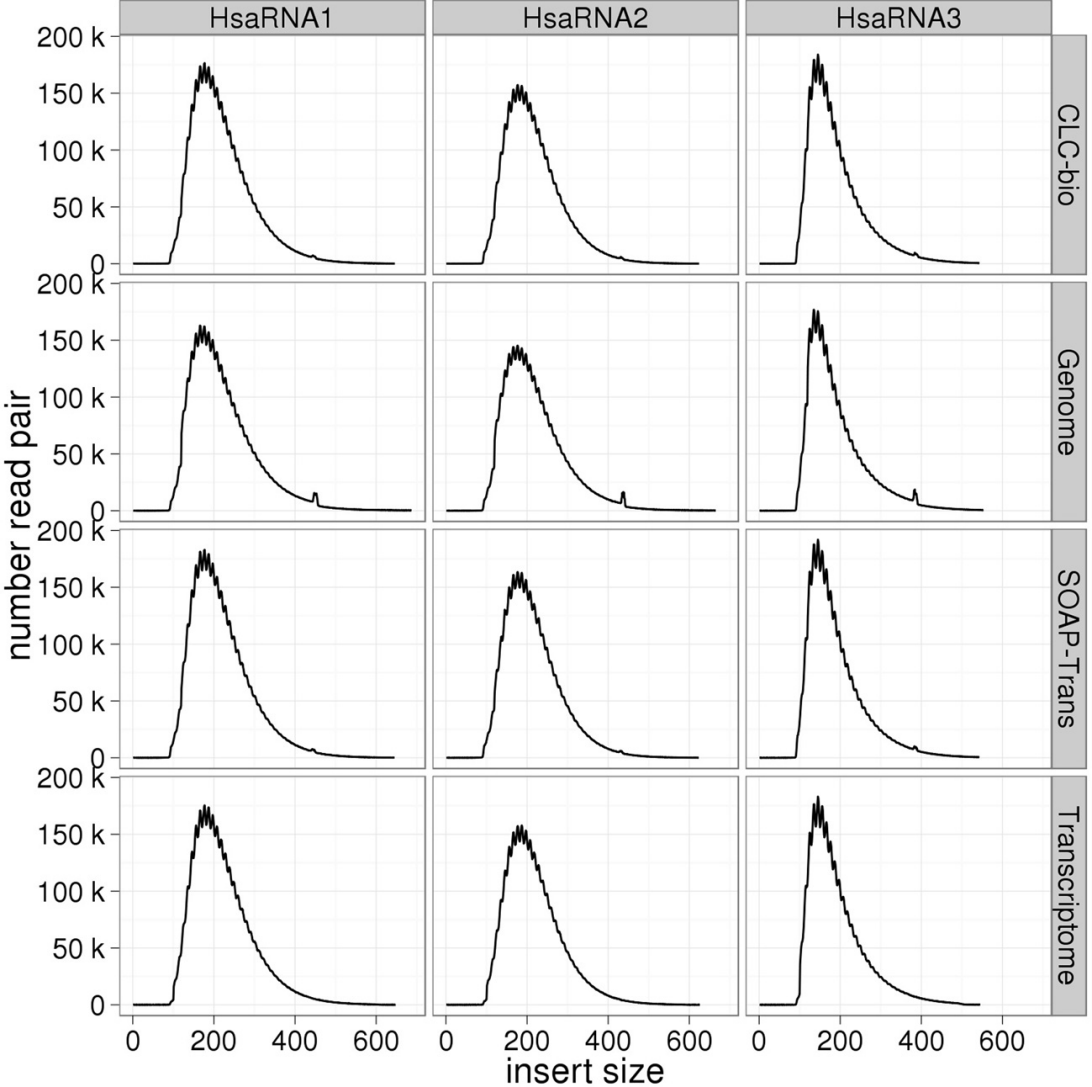
**Estimation of insert sizes for the human transcriptome libraries**. Insert sizes are plotted for each replicate library using reads mapped against *H. sapiens* reference genome and transcriptome, CLC, and SOAPdenovo2 assemblies.

Insert size distributions estimated for the genomic libraries, including the mate-pair library, against the standard reference closely matched the target, for all insert sizes and species (Figures 3–6). Distributions estimated against the draft assemblies gave very similar results. Mapping to the SOAPdenovo2 draft assemblies yielded lower numbers of mapped pairs, but gave similar insert size estimates. Similarly, insert size distributions for the RNAseq libraries were consistent across replicates and assemblies, and consistent with the reference-based estimates (Figure 7).

### Contamination check

**Figure 8:**
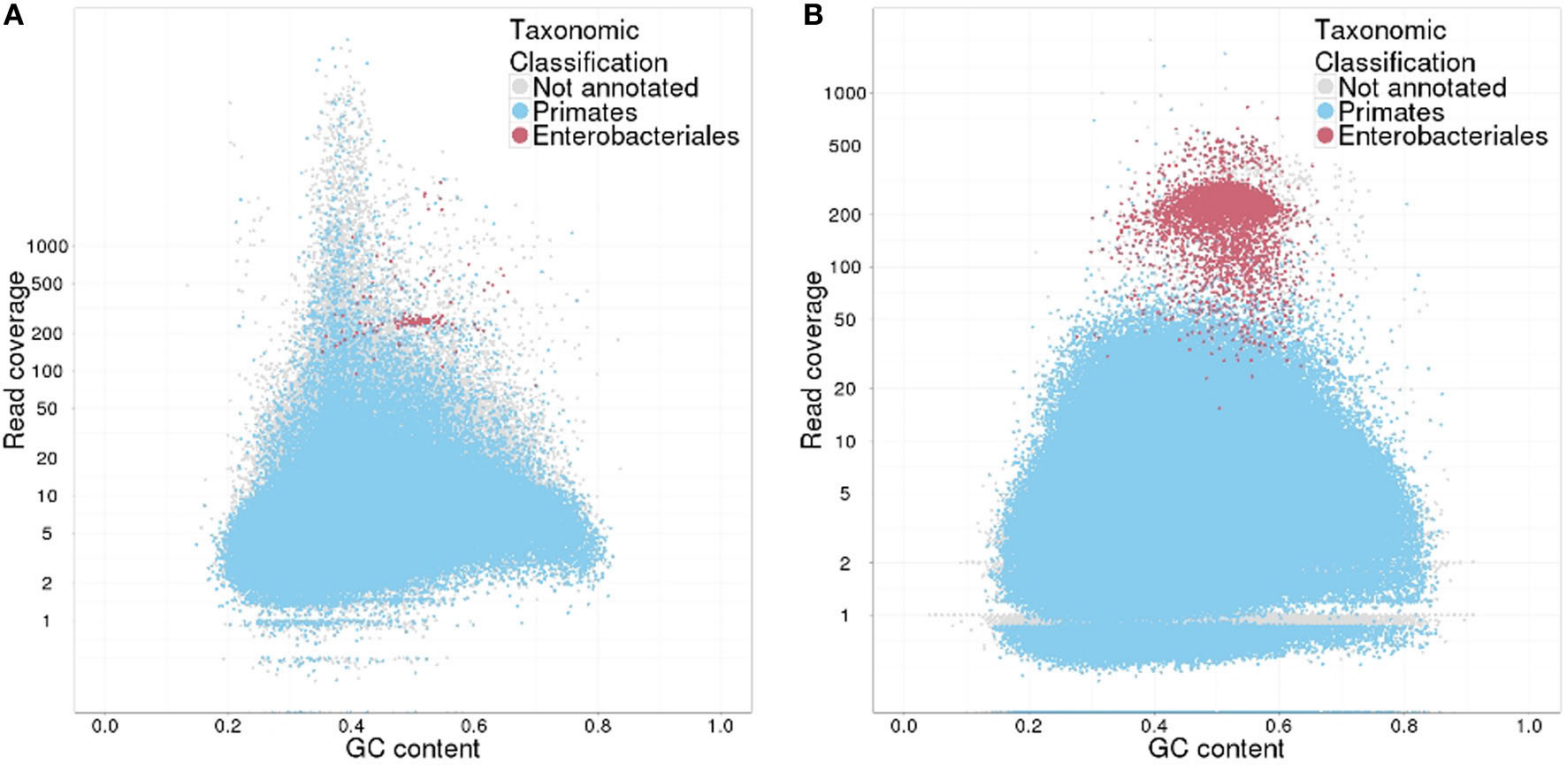
**Taxon-annotated GC-coverage (TAGC) plots for the mock-contaminated *E. coli*-human library. (A)** TAGC-plot generated after alignment to the CLC assembly; **(B)** TAGC-plot generated after alignment to the SOAPdenovo2 assembly. Individual contigs are plotted based on GC (X axis) and read coverage (Y axis, logarithmic scale). Contigs are colored according to the taxonomic order of the best megablast match to the NCBI nt database (with *E*-value < 1e-50). Contigs without an annotated BLAST match are shown in gray.

**Figure 9:**
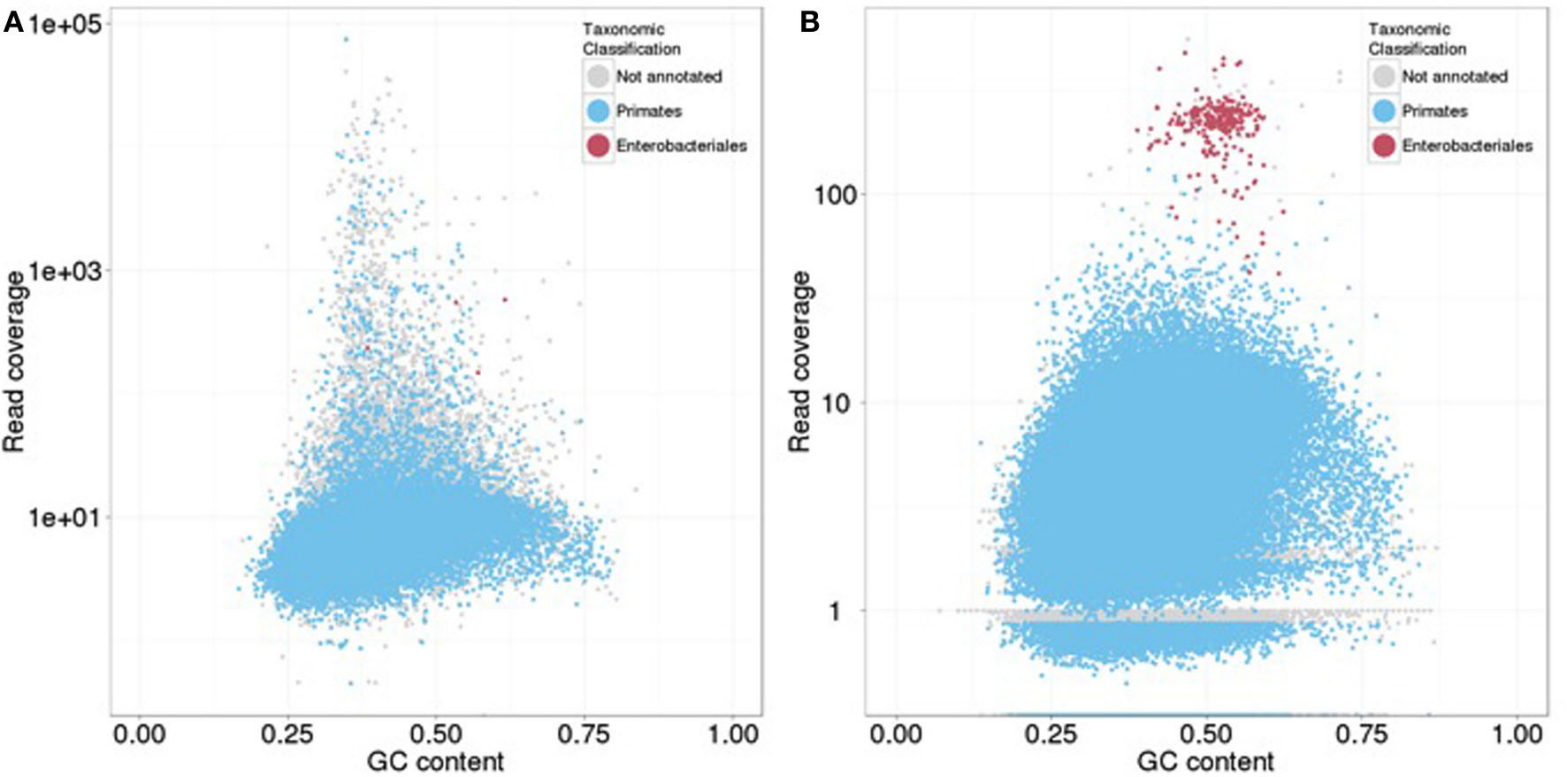
**Taxon-annotated GC-coverage (TAGC) plots from a subset of randomly selected contigs for the mock-contaminated *E. coli*-human library. (A)** TAGC-plot generated after alignment to a random subset of contigs (5) from CLC assembly; **(B)** TAGC-plot generated after alignment to to a random subset of contigs (5) from the SOAPdenovo2 assembly. Individual contigs are plotted based on GC (X axis) and read coverage (Y axis, logarithmic scale). Contigs are colored according to the taxonomic order of the best megablast match to the NCBI nt database (with *E*-value < 1e-50). Contigs without an annotated BLAST match are shown in gray.

Approximately 4% of the reads derived from the mock *E. coli*–*H. sapiens* library (EH-Mock) mapped to the *E. coli* reference (Table 5). TAGC plots generated for the CLC and the SOAPdenovo2 assemblies using all contigs revealed two clusters (Figure 8): a large cluster with read coverage between 1 and 500 and GC between 20 and 80%, and a small, well-defined cluster with coverage greater than 100 and GC between 40 and 60%. Contigs in the large cluster were annotated with BLAST matches from the taxonomic order Primates, and those in the smaller cluster were annotated with matches from the taxonomic order Enterobacteriales. Overall, ~4 and 3% of the raw reads mapped to the small cluster contigs in the CLC and SOAPdenovo2 assemblies, respectively (Table 5). TGAC plots generated from a subset of randomly selected contigs (5%) resolved SOAPdenovo2 contigs into Enterobacteriales and Primate-annotated clusters but failed to identify distinct but clusters among CLC contigs (Figure 9). 4.5% of reads mapping to the randomly selected SOAPdenovo2 contigs mapped to contigs annotated as Enterobacteriales, while this figure was only 0.04% for CLC contigs (Table 5).

**Table 5:** **Mapping statistics for the mock-contaminated *E. coli*-human genomic library**.

**Reference**	**#Sequences**	**Total bases**	**%Reference bases**	**#Reads mapped**	%Reads mapped^*^
**ALIGNMENT TO ALL CONTIGS**					
Human hg19	25	3,157,607,875	100.00	258,647,575	91.23^*^
*E.coli* K12 MG1655	1	4,705,957	100.00	11,536,466	4.07^*^
CLC all contigs	2,316,927	2,081,896,905	65.83	224,568,185	79.21^*^
CLC contigs annotated as enterobacteriales	159	4,464,778	94.88	11,514,531	4.06^*^
CLC contigs annotated as primates	953,061	649,718,210	20.58	68,061,165	24.01^*^
SOAPdenovo2 all contigs	4,548,560	2,269,464,719	71.77	192,053,787	67.74^*^
SOAPdenovo2 contigs annotated as enterobacteriales	4869	3,579,938	76.07	8,421,772	2.97^*^
SOAPdenvo2 contigs annotated as primates	3,697,548	1,848,428,560	58.54	146,728,858	51.75^*^
**ALIGNMENT TO 5% CONTIGS SELECTED AT RANDOM**					
CLC contigs in subset (5%)	115,846	104,264,699	-	15,840,263	5.59^*^
CLC contigs in subset (5%) annotated as enterobacteriales	4	2248	0.05	7073	0.04^*^
CLC contigs in subset (5%) annotated as primates	47,440	32,471,856	1.03	5,027,091	31.74^**^
SOAPdenovo2 contigs in subset (5%)	227,428	113,433,961	-	9,675,703	3.41^**^
SOAPdenovo2 contigs in subset (5%) annotated as enterobacteriales	227	184,821	3.93	435,254	4.5^**^
SOAPdenvo2 contigs in a subset (5%) annotated as primates	184,892	92,556,545	2.93	7,382,636	76.3^**^

* *Mapped reads is relative to the total number of reads generated from the library*.

** *Mapped reads is relative to the total number of reads mapped to the subset (5%) of randomly selected contigs*.

## Discussion

We have described a rapid assembly and QC protocol that permits robust estimation of a number of key QC metrics (duplication rates and library insert sizes) in the absence of a high quality reference genome. We tested the performance of this protocol by comparing QC metrics derived from analyses against the unoptimized assemblies to those derived from mapping to reference assemblies, using *E. coli*, *A. thaliana*, and *H. sapiens* raw data. Because speed is essential within a production environment, we currently use CLC to generate preliminary assemblies in our current QC pipeline. To test our strategy using open-source software, we also included SOAPdenovo2 as it is widely used and can assemble larger genomes using significantly reduced time and memory relative to other assemblers (Li et al., 2010). We recognize that other assembly tools may give different results in this context but we note that comparing our results across a range of assemblers is outside the scope of this study focused on describing methodology established and routinely used in our facility.

Despite the fact that some of the assemblies were fragmented (for example, millions of contigs for human samples), we found that QC results such as insert size and detection of contaminants derived from alignment of data to QC assemblies using CLC were equivalent to those obtained after alignment to the reference genome. In particular, insert size estimates predicted from alignment to CLC and reference genome assemblies were highly similar for both genomic (including the mate-pair library) and transcriptome libraries. These insert size frequency plots (Figures 3–6) are very helpful for general data QC, but also for directing filtering strategies to remove reads from very short inserts (a common finding in Illumina libraries generated using PCR), and in estimating parameters for full, optimal assembly. The duplicate rate estimates obtained against the reference and CLC assemblies were essentially identical across all genomic libraries. For RNAseq samples, we found that mapping to transcriptome and genome provided similar results, and that duplicate rate estimates were dependent on coverage.

The QC metrics estimated from the SOAPdenovo2 assemblies gave essentially the same results, except for the *A. thaliana* genomic libraries, where mapping rates were significantly lower than predicted by both reference-based mapping and the CLC approach. SOAPdenovo2 generated a larger number of short contigs in all assemblies, but especially for the *A. thaliana* libraries, perhaps because of features characteristic of plant genomes such as families of highly similar genes and repeats (Claros et al., 2012). As a result, many reads mapped to contig edges, remained unmapped or mapped to a different contig than their mate. Optimizing assembly parameters for SOAPdenovo2 improved mapping rates and gave estimates closer to those derived from mapping to the reference genome. Although the exact reasons for low duplicate rates assessed during mapping against the SOAPdenovo2 assemblies remain unclear, our data suggest that an excess of small contigs can lead to an underestimate of the duplicate rate.

Our contamination check protocol successfully identified the presence of exogenous reads in the mock-contaminated human library. The TAGC approach clearly identified two clusters of contigs showing contaminating Enterobacteriales sequences against a primate background in both draft assemblies. The proportion of contaminating reads estimated against the *E. coli* reference was in very close agreement with the estimate from the CLC assembly, while the same approach using SOAPdenovo2 assemblies slightly underestimated the proportions of contaminated reads. These results suggest that our protocol may also be used to quantify contamination levels, although accuracy may vary with the assembly method and the proportion of contigs used to generate TGAC plots. Significant amount of time and compute for this screening was taken by BLAST to query all contigs against NCBI nucleotide database (nt). Repeating the analysis using a subset of randomly selected contigs reduced this time to several folds and also correctly identified the presence of Enterobacteriales reads in the data but gave variable estimates of the proportions of contaminating reads, presumably due to stochastic errors due to random sampling. Thus, while sub-sampled TGAC plots may be effective to detect the presence of exogenous reads, we recommend using all contigs to maximize the power to detect and quantify contaminants.

## Conclusions

QC of raw reads is an essential first step in the analysis of NGS data. Mapping-based approaches are accurate and time efficient for collecting QC metrics such as duplicate rate and insert size, but the lack of reference sequences for non-model species has been a major bottleneck. Here, we use the power of rapid *de novo* genome and transcriptome assembly to generate contig sets to which the original reads can be mapped. The metrics derived from the unoptimized, CLC draft assembly and mapping approach are closely similar to those from reference genome mapping, and serve to deliver equivalent QC data. While our approach successfully estimated the insert size distribution of a 3 kb mate pair library prepared from *E. coli*, we recognize that mate-pair libraries can be challenging to assemble, especially when the virtual insert size is large and/or the target genome is complex. These will typically be generated alongside a range of standard libraries with different insert sizes. In practice we recommend to map the reads derived from the mate pair library against the draft assembly of contigs generated from the standard libraries and calculate an estimate of insert length and duplicate rate from this alignment.

The use of SOAPdenovo2 as an alternative assembler was generally successful and gave similar metrics to CLC in most cases. However, this was not true for predicting the duplicate rate. Assembling difficult genomes such as those of plants can lead to an underestimate of the true duplicate rate. In this case, some parameter optimization (e.g., k-mer size) can help in generating more robust QC metrics. While this approach is likely to be impractical in a production environment where different libraries may require different assembly parameters, other assemblers may perform better in this context and further work is needed to identify suitable alternatives to CLC.

We recommend GC, coverage and BLAST-based similarity screening of preliminary assemblies for exclusion of contaminating data before continuing with downstream analyses. This is easily achieved through the use of TAGC plots. For contamination check, we used all contigs as input to the TAGC pipeline. Random selection of contigs can be useful to speed up the process of screening for contaminants but may significantly reduce the power to obtain quantitative estimates of contaminating reads.

## Author contributions

Karim Gharbi, Mark Blaxter and Urmi H. Trivedi designed the study. Anna Montazam and Jenna Nichols prepared the sequencing libraries. Urmi H. Trivedi drafted the manuscript and carried out data analysis with support from Timothée Cézard, Stephen Bridgett, Karim Gharbi, and Mark Blaxter. All authors contributed to the manuscript.

### Conflict of interest statement

The authors declare that the research was conducted in the absence of any commercial or financial relationships that could be construed as a potential conflict of interest.

## Footnotes

^1^FASTX-Toolkit. [Online]. Available online at: http://hannonlab.cshl.edu/fastx_toolkit/

^2^PICARD. [Online]. Available online at: http://picard.sourceforge.net/

^3^CLC-bio assembly-cell. [Online]. Available online at: http://www.clcbio.com/
